# Epigenetics as a mechanism driving polygenic clinical drug resistance

**DOI:** 10.1038/sj.bjc.6603024

**Published:** 2006-02-21

**Authors:** R M Glasspool, J M Teodoridis, R Brown

**Affiliations:** 1 Centre for Oncology and Applied Pharmacology, Glasgow University, CRUK Beatson Laboratories, Garscube Estate, Glasgow G61 1BD, UK

**Keywords:** chemotherapy, biomarkers, DNA methylation, drug resistance, epigenetics

## Abstract

Aberrant methylation of CpG islands located at or near gene promoters is associated with inactivation of gene expression during tumour development. It is increasingly recognised that such epimutations may occur at a much higher frequency than gene mutation and therefore have a greater impact on selection of subpopulations of cells during tumour progression or acquisition of resistance to anticancer drugs. Although laboratory-based models of acquired resistance to anticancer agents tend to focus on specific genes or biochemical pathways, such ‘one gene : one outcome’ models may be an oversimplification of acquired resistance to treatment of cancer patients. Instead, clinical drug resistance may be due to changes in expression of a large number of genes that have a cumulative impact on chemosensitivity. Aberrant CpG island methylation of multiple genes occurring in a nonrandom manner during tumour development and during the acquisition of drug resistance provides a mechanism whereby expression of multiple genes could be affected simultaneously resulting in polygenic clinical drug resistance. If simultaneous epigenetic regulation of multiple genes is indeed a major driving force behind acquired resistance of patients' tumour to anticancer agents, this has important implications for biomarker studies of clinical outcome following chemotherapy and for clinical approaches designed to circumvent or modulate drug resistance.

With the increasing variety of options for the treatment of cancer, it is becoming essential that the choice of anticancer therapy, or optimal combination of therapies, is based not only on conventional clinical/pathological criteria but also on the molecular phenotype of the tumour. Many solid tumours are initially sensitive to chemotherapy, but the vast majority will recur or progress with ultimate failure of conventional cytotoxic chemotherapy treatment. In general, novel experimental therapies are first examined for efficacy in patients that have failed standard treatments and whose tumours have acquired resistance to cytotoxic drugs. The pattern of gene expression of a tumour that no longer responds to conventional treatment will be very different from that of the tumour at presentation due to selection of drug-resistant subpopulations. However, we know very little about the molecular characteristics of tumours after conventional treatment failure or the underlying mechanisms that drive the acquisition of drug resistance (Agarwal and Kaye, 2003).

Laboratory-based studies have identified a wide variety of biochemical pathways and many hundred genes that can potentially influence response to treatment in tumour cells. Early work in drug resistance identified genes such as MDR1 (P-glycoprotein) (Gottesman, 1993) and p53 (Lowe *et al*, 1993) as crucial in determining drug resistance in experimental models of *in vitro* cell lines or transgenic mice. However, there is relatively little evidence that, individually, these mechanisms are able to predict treatment outcome in a manner that is comparable to known prognostic markers such as stage, performance status and histological grade (Agarwal and Kaye, 2003; Hall *et al*, 2004). The variability in quality of prognostic and predictive biomarker studies can make reaching a consensus on the value of a given marker challenging and recent recommendations have emphasised the need for appropriate design and reporting of biomarker studies (http://www.cancerdiagnosis.nci.nih.gov/assessment/progress/progress/remark.html). Furthermore, response to treatment is only one factor influencing clinical outcome, numerous other tumour characteristics, such as capacity for invasion/metastasis or escape from the immune response, will also have an impact and may do so irrespective of the therapies used, diluting any association between a marker of drug resistance and clinical outcome. For instance, in the case of ovarian cancer, one of the strongest prognostic markers associate with time to progression of a tumour after treatment is the number of infiltrating T cells (Zhang *et al*, 2003). However, while these factors may confound the analysis of drug-resistance mechanisms, it is also becoming apparent that ‘one gene : one outcome’ is an oversimplification for acquired resistance to treatment of cancer patients. Thus, it seems increasingly likely that clinical drug resistance is due to polygenic expression changes involving multiple mechanisms rather than to the alteration of a single pathway or gene.

An analogy can be made between clinical drug-resistance genes and cancer susceptibility genes. Cancer susceptibility genes such as retinoblastoma (*RB1*) and adenomatois polyposis coli (*APC*) were originally identified as rare, mutant alleles that significantly increase the risk of cancer when inherited through the germ line. More recently, it has been argued that the greater part of cancer predisposition may be due to a combination of weak genetic variants at many different loci rather than to single high penetrance genes (Balmain *et al*, 2003). Similarly, the combination of weak effects on drug resistance due to expression changes at many genes may be more significant than the effect of any single gene. Since most cytotoxic drugs have a low therapeutic index, additive effects of multiple low fold changes in drug resistance may be sufficient to cause clinical treatment failure. However, identification and evaluation of multiple, small additive effects on clinical outcome following chemotherapy will require robust and novel statistical and computational approaches that allow nonrandom clustering of effects to be identified. In order to avoid the pitfalls inherent in analysing high-dimensional data sets such as multiple testing and limited sample size, large-scale prospective clinical studies are required. In addition, it may be more informative to study tumours longitudinally, as they acquire resistance during treatment rather than simply sampling tumours at presentation and to use surrogate end points more specific to drug resistance, such as response rather than overall survival.

## GENETIC *VS* EPIGENETIC ALTERATIONS OF RESISTANCE GENES

At the time of writing, we have been unable to identify any study of clinical material that has identified acquisition of a *p53* mutation during treatment of a given patient and similarly gene amplification of *MDR1*, although widely observed in highly resistant cell lines, is only rarely observed following chemotherapy. Therefore, although mutations in genes such as *MDR1* and *p53* confer drug resistance *in vitro* and in animal models, and they may have a role in inherent resistance, there is little evidence that such genetic changes have a role in acquired clinical resistance following anticancer therapy.

It is clear that changes in gene expression do occur following chemotherapy leading to the question, if not gene mutations, what are the mechanisms leading to changes in gene expression? The answer may lie in the increasing evidence that epigenetic changes can be a crucial driving force behind the acquisition of drug resistance (Teodoridis *et al*, 2004). Indeed studies of drug-resistant cell line models have shown that multiple changes in methylation of CpG islands and epigenetic regulation occur following drug selection (Wei *et al*, 2003).

Epigenetics changes are heritable changes in gene expression that do not involve an alteration in the DNA sequence. Within the nucleus, DNA is packaged, together with histone proteins, into a higher order structure known as chromatin. Interpretation of genetic information coded within the DNA is regulated by mechanisms that involve stable and heritable modifications of DNA and histones. These modifications include methylation of DNA at CpG dinucleotides and methylation, acetylation and phosphorylation of histones. Changes in the patterns of these modifications are associated with chromatin remodelling and can result in changes in gene expression through increasingly understood mechanisms (Lachner *et al*, 2003).

DNA methylation involves the transfer of a methyl group to the carbon-5 position of cytosine residues, and occurs almost exclusively at cytosines that are followed by a guanine (CpG dinucleotides). CpG dinucleotides are relatively rare in the bulk of the genome and are nearly always methylated, but small stretches of DNA occur that are rich in CpG dinucleotides, so called CpG islands. These are usually unmethylated in normal cells and are often associated with the promoter regions of genes (Hendrich and Bird, 2000). Methylation of cytosines within these islands is associated with binding of methyl-binding domain (MBD) proteins, recruitment of histone deacetylases (HDACs) and histone methyltransferases, histone modification, chromatin condensation and transcriptional inactivation of the associated genes. A large number of genes where aberrant methylation of CpG islands within their promoters is associated with gene inactivation have now been identified in tumours (for methods of analysing CpG island methylation, see Box 1). These include genes involved in all aspects of tumour development and also in response to treatment (Teodoridis *et al*, 2004). Furthermore, for many genes such as *hMLH1*, *BRCA1* and *E-CADHERIN*, aberrant methylation of CpG islands is a far more frequent mechanism of gene inactivation in sporadic tumours than gene mutation or deletion.

Gene inactivation by DNA methylation can occur at a rate several orders of magnitude higher than inactivation of the same gene by mutation (Bhattacharyya *et al*, 1994). So, if inactivation of a gene is an important mechanism driving the acquisition of drug resistance, the probability of this occurring by methylation and being selected for during chemotherapy is much more likely than it occurring by mutation. It has also been suggested that some tumours may acquire a CpG island methylator phenotype, that is, concurrent methylation of genes occurring in a nonrandom manner (Toyota *et al*, 1999). Cellular acquisition of a methylator phenotype could give cells a higher probability of cell transformation during carcinogenesis, as has been proposed for gene mutations and the mutator phenotype (Loeb, 1994). Disruption of the cellular processes involved in methylation could lead to concurrent hypermethylation of multiple genes, including tumour suppressor genes, and as a result lead to oncogenic transformation. A possible consequence of this would be that in a tumour with a methylator phenotype there would also be a higher probability of multiple drug-resistance/sensitivity genes becoming methylated, with associated changes in gene expression. Thus, epigenetic silencing may occur fortuitously during tumour development and only confer an advantage to tumour cells when they are treated with chemotherapy or radiotherapy. However, the existence of a distinct methylator phenotype has been challenged, since a bimodal distribution of methylation frequency has not been seen in the same way as observed for gene mutation in tumour cells with the mutator phenotype (Yamashita *et al*, 2003; Anacleto *et al*, 2005).

Nevertheless, the vast majority of tumours, if not all, have aberrant DNA methylation at CpG islands and epigenetic silencing of the associated genes. Patterns of CpG island methylation differ between and within tumour types in a manner that suggests that methylation is not a random process (Costello *et al*, 2000; Esteller *et al*, 2001; Wei *et al*, 2002). Epigenetic inheritance of transcription patterns has been implicated in the control of cell proliferation during development, as well as in stem-cell renewal and cancer (Valk-Lingbeek *et al*, 2004). However, the mechanisms and selective processes that give rise to specific methylation patterns in tumours remain unclear and are likely to be complex. Changes in cell metabolism (Paz *et al*, 2002), ‘epigenetic drift’(Egger *et al*, 2004) and ageing (Richardson, 2002) have all been proposed. For instance, there is a global decrease in global 5 methlycytosine levels in DNA as cells age which is similar to that observed in many tumours (Richardson, 2002). At the same time, localised hypermethylation occurs at some CpG islands. In a restriction landmark genome scanning study of CpG island methylation in T lymphocytes from newborn, middle age and elderly subjects, only 29 of more than 2000 loci examined were found to alter methylation with ageing, with 23 increasing methylation, and six decreasing. The same subset also changed methylation status with age in the oesophagus, lung and pancreas, but in variable directions (Tra *et al*, 2002). Thus, age-specific methylation also occurs in a nonrandom manner suggesting a tightly controlled process. What ever the process, it seems likely that epigenetic changes regulating gene expression offer a more rapid means by which tumour cells can adapt to new environment such as cytotoxic drug therapy than genetic change and because such changes are heritable they can be passed on to daughter cells without the need for continuous selection pressure producing persistent acquired resistance.

## EVIDENCE FOR THE ROLE OF EPIGENETIC MECHANISMS IN DRUG RESISTANCE

Altered expression of genes involved in apoptosis and DNA repair may play an important role in determining response to treatment and there are many examples of such genes being methylated in tumours (see Table 1). However, methylation of individual genes may have opposing effects on drug sensitivity. For instance, methylation of DNA repair genes such as *MGMT* and *FANCF* may lead to inactivation of DNA repair and confer chemosensitivity, while methylation and epigenetic silencing of proapoptotic genes such as *hMLH1* and *APAF1* would confer resistance (Esteller *et al*, 2000; Soengas *et al*, 2001; Taniguchi *et al*, 2003; Teodoridis *et al*, 2004).

The DNA mismatch repair protein, hMLH1, has been shown to be necessary for engagement of a variety of downstream cellular responses to alkylating agents and cisplatin-induced DNA damage (Papouli *et al*, 2004). Re-expression of hMLH1 in isogenic model systems has demonstrated that loss of hMLH1 expression confers resistance to alkylating agents and cisplatin. The frequency of *hMLH1* methylation in ovarian tumours increases after chemotherapy (Strathdee *et al*, 1999). Tumours frequently release DNA which can subsequently be isolated from plasma samples (Johnson and Lo, 2002). Genetic and epigenetic changes that are present in the tumour can be detected in tumour DNA isolated from plasma. Analysis of *hMLH1* methylation in tumour DNA isolated from plasma of patients with ovarian cancer before chemotherapy and at relapse showed that 25% of patients acquired *hMLH1* methylation during chemotherapy and acquisition of *hMLH1* methylation was independently associated with poor overall survival, potentially as a result of poor response to subsequent lines of chemotherapy (Gifford *et al*, 2004).

In contrast to proapoptotic genes, loss of expression of DNA repair genes may be associated with increased sensitivity to chemotherapy. The DNA repair enzyme MGMT (O6 methyl guanine methyltransferase) removes mutagenic alkyl-groups from the O6-position of guanine, which could otherwise lead to G → A transitions after DNA replication (Gerson, 2004). As a result, it inhibits the killing of tumour cells by alkylating agents. Hypermethylation of the *MGMT* promoter and associated loss of expression correlates with response to temozolamide and BCNU in primary gliomas (Esteller *et al*, 2000; Paz *et al*, 2004) and is an independent predictor of overall and progression-free survival in patients with diffuse large B-cell lymphoma treated with cyclophosphamide-containing regimens (Esteller *et al*, 2002). Importantly, the methylation status of MGMT in gliomas at presentation does not correlate with the clinical response when temozolamide is used at relapse, demonstrating that the value of biomarkers may depend on when during tumour progression or treatment they are measured.

There is thus growing evidence that CpG island methylation of genes with a known direct involvement in drug responses has a potential role in predicting clinical outcome following chemotherapy. However, there is a need for studies to investigate the potential to use methylation patterns of known or unknown genes to identify which patients may benefit from particular chemotherapeutic regimes or biological therapies. Given the potential of opposing effects depending on which genes are methylated, it is vital to examine whether particular methylation events are dominant in conferring resistance. Methods that allow genome-wide analysis of methylation patterns may be particularly important for these types of study (Box 1). In a study of late-stage ovarian tumours, increased methylation of a subset of CpG islands significantly correlated with worse clinical outcome, as defined by the time to clinical disease recurrence after chemotherapy (Wei *et al*, 2002). However in a study of 106 stage III/IV ovarian cancers, methylation of at least one of a group of genes involved in DNA repair/drug detoxification (*BRCA1*, *GSTP1*, *MGMT*) was associated with improved response to chemotherapy (Teodoridis *et al*, 2005).

Large-scale analysis of methylation patterns and correlation with response is intrinsically susceptible to the problems of multiple testing. This can be reduced by grouping genes into predefined groups according to a biological hypothesis such as grouping those with similar biological roles or within the same pathway, on the assumption that disruption of any one gene within a pathway or group will disrupt the functioning of that cellular response. This is undoubtedly an oversimplification and the approach will need to be refined as more sophisticated molecular interaction maps and networks are developed (Pommier *et al*, 2004). An alternative approach will be to use supervised search algorithms that efficiently search array data to identify clusters that associate with clinical outcome (for instance, see Bair and Tibshirani, 2004).

## OVERCOMING EPIGENETIC RESISTANCE MECHANISMS

Epigenetic modifications require active mechanisms of maintenance and so unlike genetic modifications, they are amenable to pharmacological manipulation. 5-Azacytidine and its deoxyribose analogue, 5-aza-2′-deoxycytidine (decitabine), have been used for many years to inhibit DNA methyltransferases and reverse DNA methylation in tissue culture (Brown and Plumb, 2004). These demethylating agents have been shown to reactivate expression of numerous methylation-silenced genes. Decitabine has clinical activity as a single agent in myelodysplastic syndrome (MDS), CML and AML (Issa *et al*, 2004). Its activity in solid tumours as a single agent has so far been disappointing. However, it may have a role in sensitising tumours to other anticancer therapies by causing re-expression of genes involved in drug sensitivity (Plumb *et al*, 2000). *In vitro* the differentiating effect of decitabine in cultured fibroblasts has a narrow dose window with a loss of action at high doses possibly caused by cytotoxicity as a result of its incorporation into DNA (Taylor and Jones, 1979). It may, therefore, be more appropriate to use demethylating agents at concentrations below the maximally tolerated dose, but still at a level where they are known to cause demethylation and induce gene re-expression. Consistent with this, a low dose schedule appeared to be superior to schedules using higher doses in a study of haematological malignancies (Issa *et al*, 2004). This has the advantage of reducing the bone marrow toxicity of decitabine and making it easier to combine it with conventional cytotoxics. Histone deacetylase activity is important in the transcriptional repression of methylated sequences (Fischle *et al*, 2003). The combination of DNA-demethylating agents and HDAC inhibitors causes synergistic re-expression of epigenetically silenced genes (Cameron *et al*, 1999). It also produces synergistic antitumour effects and increased sensitivity to chemotherapeutic agents in cell line models (Boivin *et al*, 2002). The potential of this approach is now being assessed in clinical trials (http://www.clinicaltrials.gov/ct/show/NCT00114257).

Histone deacetylase inhibitors and demethylating agents, such as decitabine, will affect the expression of multiple genes. Given the potential for opposing effects on chemosensitivity when different genes are re-expressed, it could be argued that we need to develop epigenetic therapies that are more gene specific in their mechanism of action. However, if we consider drug resistance to be a polygenic process, then there may be advantages to a multitargeted approach. This implies that some patients may benefit from epigenetic therapies as chemosensitisers, while others will not or may even do worse. Therefore, it will be vital to identify patterns of methylation that reliably predict for response to treatment and whether particular methylation events are dominant in conferring resistance. In order to do this we need robust clinically applicable technology to determine methylation patterns in tumours both at presentation and at relapse. There is also a need for pharmacodynamic markers of response to demethylating agents. Demethylation can be monitored on a whole-genome level or by analysis of individual genes (Lyko and Brown, 2005). It has been shown that genomic DNA methylation levels are decreased in peripheral blood mononuclear cells from xenograft tumour-bearing mice treated with 5-aza-2′-deoxycytidine (Plumb *et al*, 2000). This decrease closely coincided with the demethylation of the *hMLH1* promoter in the tumours, which indicates that peripheral blood can serve as a surrogate tissue for determining pharmacodynamic characteristics of DNMT inhibitors. However, although demethylation of individual genes such as *p15* has been demonstrated in clinical trials (Daskalakis *et al*, 2002), the prognostic value of these methylation changes remains to be established.

## CONCLUSIONS

Aberrant epigenetic regulation, such as DNA methylation of CpG islands, occurs at many genes and in all cancers. CpG island methylation is a potentially important driving force both for tumorogenesis and for drug resistance. The use of demethylating agents and HDAC inhibitors offers the potential to favourably alter the gene expression profile of tumours to cause tumour cell death and increased apoptotic response to established cytotoxic agents. However, we need to identify and evaluate in greater detail the epigenetic characteristics of tumours that predict for lack of response to conventional treatment, so as to identify those patients who may particularly benefit from an epigenetic approach. The polygenic nature of these changes will make this challenging. Towards this objective, genome-wide CpG island methylation of patients' tumours can be examined, as well as detailed characterisation of methylation of individual CpG islands. Examination of tumour DNA released into body fluids may make the large numbers required for these analyses more feasible. These assays need to be conducted in an appropriate quality assured manner and their utility properly evaluated in prospective, randomised trials. Although the epigenetic therapies now undergoing clinical evaluations show promise, there is a need for further agents, which are more specific for epigenetic targets. This need not equate to more gene specificity, but rather to less nonspecific toxic effects such as the myelosuppression seen with decitabine which may be the result of direct cytotoxic effects of decitabine rather than demethylation. The clinical development of epigenetic therapies will require the development of surrogate pharmacodynamic markers to assess whether these therapies are having their desired pharmacodynamic effect (e.g. global or gene-specific demethylation) and then whether this translates into clinical benefit. Epigenetic pharmacodynamic markers can be used as novel end points in early clinical trials allowing recommended doses to be based on maximal biological effect rather than maximum tolerated dose. Thus, such pharmacodynamic and predictive epigenetic biomarkers, together with targeted drug development, will allow rational and efficient evaluation of novel epigenetic therapies for cancer treatment.

## Figures and Tables

**Box 1:** Methods for detecting CpG island methylation

Methods for the analysis of CpG-island methylation are available both genome-wide and at the single gene level. Restriction landmark genomic scanning (RLGS) is performed by digesting genomic DNA with a methylation-sensitive restriction enzyme, end labelling of the resulting DNA fragments and subsequent digestion with two different restriction enzymes and 2-dimensional gel electrophoresis (Costello *et al*, 2000). Comparison of signal intensities between tumour and normal DNA after autoradiography allows estimation of the number of aberrantly methylated CpG islands in tumours, and individual aberrantly methylated CpG islands can be identified by sequencing. Differential methylation hybridisation (DMH) is an alternative means of examining genome-wide methylation patterns that uses restriction digestion of genomic DNA and ligation to linkers (Huang *et al*, 1999), followed by digestion with a methylation-sensitive restriction enzyme such as *Bst*UI, PCR amplification and hybridisation to CpG-rich DNA sequences (representing putative CpG islands). Comparison to hybridisation signals obtained from undigested linker-ligated DNA allowed the identification of aberrantly methylated CpG islands. Methylation sensitive-representational difference analysis (MS-RDA) uses genomic tester and driver DNA samples digested with the methylation-sensitive restriction enzyme *Hpa*II (Ushijima *et al*, 1997). Sequences that are specific for the tester amplicon are subsequently enriched by repeated cycles of subtractive hybridisations.

Several methods for the analysis of the methylation status of individual CpG islands utilise bisulphate treatment of DNA, which has been described in detail (Grunau *et al*, 2001; Warnecke *et al*, 2002). Bisulphite treatment of DNA converts unmethylated cytosines into uracil but does not affect methylated cytosines. A difference in methylation is thus converted into a difference in sequence. A widely used method for analysing the methylation status of specific sequences is methylation-specific PCR (MSP) (Herman *et al*, 1996). Methylation-specific PCR is performed using primers specific for either unmethylated or methylated sequences, thereby allowing the detection of the respective methylation state. Among the advantages of MSP are its easy detection due to its gain-of-signal character and its high sensitivity, allowing the detection of as little as 0.1% methylation in a DNA sample (Herman *et al*, 1996). The MethyLight technique also involves bisulphite modification. Fluorescence-based PCR is then performed with primers that either overlap CpG methylation sites or that do not overlap any CpG dinucleotides. Sequence discrimination can occur either at the level of the PCR amplification process or at the level of the probe hybridisation process or both (Eads *et al*, 2000). Combined restriction analysis (COBRA) uses primers that amplify the template irrespective of its methylation state (Xiong and Laird, 1997). The PCR product should therefore be heterogeneous and reflect the various methylation states present in the template. Discrimination of methylation states is achieved by restriction digest using a restriction site whose presence after bisulphite modification depends on the methylation state of the DNA. Combined restriction analysis allows the quantification of the methylation, but its disadvantage is that the methylation of one CpG site is not necessarily representative for the other CpG sites in the analysed sequence. The highest accuracy of methylation density in a region of DNA is achieved by bisulphite sequencing. As in COBRA, the modified DNA is amplified irrespective of its methylation state, but subsequently the amplicon is subcloned and sequenced. This not only allows detection of methylation with a single-nucleotide resolution but also gives information about the distribution of methylated cytosines within individual DNA molecules. The disadvantage is that bisulphite sequencing is relatively labour-intensive.

**Table 1:** Examples of genes associated with drug resistance

**Gene**	**Function**	**Evidence for role in drug sensitivity**	**Reference**
Apaf 1	Proapoptotic, binds and promotes caspase 9 activation	Methylation in melanoma cells can be reversed by DNMT inhibitors and this is associated with increased sensitivity to doxorubicin	Soengas *et al* (2001)
Caspase 8	Proapoptotic	Frequently methylated in tumours. Reversal of methylation associated with increased sensitivity to doxorubicin, etoposide and cisplatin in Ewings sarcoma, neuroblastoma, medulloblastoma and melanoma cell lines	Fulda *et al* (2001)
hMLH1	DNA mismatch repair protein	Methylation and loss of expression associated with resistance to cisplatin in cell lines, which can be reversed by demethylation with decitabine. Increased frequency of methylation after chemotherapy. Acquisition of *hMLH1* methylation during chemotherapy is independently associated with poor overall survival in ovarian patients	Gifford *et al* (2004)
FancF	Activates DNA repair complex containing BRCA1, and BRCA2 loss cause a decreased ability to repair chemotherapy-induced damage	Methylation observed in cells with a defective BRCA2 pathway and increased sensitivity to cisplatin. Demethylation of *FANCF* with decitabine reduced sensitivity towards cisplatin in these cell line models	Taniguchi *et al* (2003)
MGMT	Removes mutagenic alkyl-groups from the O6-position of guanine	Methylation and associated loss of expression correlates with response to temozolamide and BCNU in primary gliomas and overall and progression-free survival in patients with diffuse large B-cell lymphoma treated with cyclophosphamide-containing regimens	Paz *et al* (2004)
MCJ	Unknown	Methylation associated with poor response to therapy and poor overall survival in ovarian patients	Strathdee *et al* (2005)
ER*β*		Methylated in 50% of invasive breast cancers. Methylation of *ERβ* less frequent and expression rate was higher in tamoxifen-resistant compared to control tumours	Chang *et al* (2005)
